# Tadalafil: Protective Action against the Development of Multiple Organ Failure Syndrome

**DOI:** 10.21470/1678-9741-2017-0503

**Published:** 2017

**Authors:** Granville G. de Oliveira, Samer A. H. de Oliveira, Paulo Henrique H. Botelho, Marcos Aurelio Barboza de Oliveira, Ka Bian, Ferid Murad

**Affiliations:** 1 Universidade Católica de Brasília (UCB), Brasília, DF, Brazil; 2 Faculdade de Medicina de São José do Rio Preto (FAMERP), São José do Rio Preto, SP, Brazil; 3 George Washington School of Medicine and Health Sciences, Washington, DC, USA; 4 University Hospitals Case Medical Center, Department of Cardiology, Division of Electrophysiology, Cleveland, OH, USA; 5 Centro Universitário de Votuporanga (Unifev), Votuporanga, SP, Brazil; 6 Department of Biochemistry and Molecular Biology. George Washington School of Medicine and Health Sciences, Washington, DC, USA

**Keywords:** Multiple Organ Failure, Nitric Oxide, Phosphodiesterase Inhibitors, Systemic Inflammatory Response Syndrome

## Abstract

**Introduction:**

Multiple organ failure syndrome (MOFS) is a pathology associated to unspecified and severe trauma, characterized by elevated morbidity and mortality. The complex inflammatory MOFS-related reactions generate important ischemia-reperfusion responses in the induction of this syndrome. Nitric oxide elevation, through the activation of cyclic guanosine monophosphate (cGMP), has the potential of counteracting the typical systemic vasoconstriction, and platelet-induced hypercoagulation. Tadalafil would possibly act protectively by reducing cGMP degradation with consequent diffuse vasodilatation, besides reduction of platelet-induced hypercoagulation, thus, preventing multiple organ failure syndrome development.

**Methods:**

The experimental protocol was previously approved by an institution animal research committee. Experimental MOFS was induced through the stereotaxic micro-neurosurgical bilateral anterior hypothalamic lesions model. Groups of 10 Wistar rats were divided into:

a) Non-operated control;
b) Operated control group;
c) 2 hours after tadalafil-treated operated group;
d) 4 hours after tadalafil-treated operated group;
e) 8 hours after post-treated operated group. The animals were sacrificed 24 hours after the neurosurgical procedure and submitted to histopathologic examination of five organs: brain, lungs, stomach, kidneys, and liver.

**Results:**

The electrolytic hypothalamic lesions resulted in a full picture of MOFS with disseminated multiple-organs lesions, provoked primarily by diffusely spread micro-thrombi. The treatment with tadalafil 2 hours after the micro-neurosurgical lesions reduced the experimental MOFS lesions development, in a highly significant level (P<0.01) of 58.75%. The treatment with tadalafil, 4 hours after the micro-neurosurgically-induced MOFS lesions, also reduced in 49.71%, in a highly significant level (P<0.01). Finally, the treatment with tadalafil 8 hours after the neurosurgical procedure resulted in a statistically significant reduction of 30.50% (P<0.05) of the experimentally-induced MOFS gravity scores.

**Conclusion:**

The phosphodiesterase 5 inhibitor, tadalafil, in the doses and timing utilized, showed to protect against the experimentally-induced MOFS.

**Table:** 

Abbreviations, acronyms & symbols				
AH	= Anterior hypothalamus		GnRH	= Gonadotrofin-releasing hormone
AR	= Alarm reaction		ICU	= Intensive care unit
cGMP	= Cyclic guanosine monophosphate		MOF	= Multiple organ failure
CGS	= Consolidated gravity scores		MOFS	= Multiple organ failure syndrome
CNS	= Central nervous system		NO	= Nitric oxide
EDRF	= Endothelium-derived relaxing factor		NOS	= Nitric oxide synthase
EL	= Electrolytic lesions		PDE5	= Phosphodiesterase 5

## INTRODUCTION

Trauma is a factor of unquestionable epidemiologic importance in modern industrial societies, especially in the last hundred years^[1]^. In United States, trauma is the leading cause of morbidity and mortality for persons, from birth to 44 years of age. In the last decade, more than 13 million adults with 20-49 years of age were injured, and nearly 80,000 were killed, with US$ 406 billion in lost productivity and medical bills every year^[2]^. In this scenario, the prevalence of multiple organ failure syndrome (MOFS) has been very high, affecting one-third of all in-hospital patients, being responsible for more than 50% of all intensive care unit (ICU) patients' deaths^[3]^. This previously unknown post-trauma sequential dysfunction syndrome, despite being suspected by medical teams, since the ICU emergence, in 1958, was only officially recognized when the global term "multiple organ failure" (MOF) first appeared in Index Medicus, in 1983^[1]^. However, its existence could be found in very remote descriptions, like the one, in 1823, of William Cumin^[3]^, surgeon of the Royal Infirmary and the Asylum of Lunatics, of Glasgow, and by Joseph Swan, physician of the Lincoln County Hospital, in the same number of the Edinburgh Medical and Surgical Journal. They reported the pathological lesions in several organs not primarily damaged of six burned patients that died days after trauma.

Cumin^[4]^ observed, with high accuracy: "...distant internal parts sympathized with the burned surface and suffer inflammation...". Other XIX century authors, such as Cooper^[5]^, in 1839; Long^[6]^, in 1840; or Curling^[7]^, in 1842, among others, had described similar findings, with no repercussion in the scientific community.

Recent authors had described similar pathological findings secondarily associated with trauma, in soldiers wounded in military actions during the World War II, as Moon^[8]^, in 1947, and Mallory et al.^[9]^, in 1950. These papers described the occurrence of a picture of global and diffuse organic deterioration, typical of MOF, an unknown pathology at that time. They reported the presence of diffuse endothelial lesions, in several organs, with capillary and venules engorgement. The development of disseminated micro-thrombosis was a most common finding. In 85% of the cases, the examined lungs showed edema and congestion. Atelectasis could be found in 70%, and fat emboli in 65% of the cases. Intra-alveolar hemorrhages were found in 51% of all cases. The cardiac histopathology showed fat vacuolization in 24 of 44 (54.5%) examined cases. The liver examination presented fat vacuolization in 53 of 60 (88.3%) examined cases in patients that died up to 96 hours after trauma. In these cases, were detected the presence of centrilobular pyknosis and nuclear disintegration. In these studies, kidneys microscopy showed presence of fat vacuolization, especially of the ascending limb of loop of Henle, in 55% of cases, reaching 85% when death occurred between the first and fourth post-trauma day. Tubular epithelial necrosis, basal membrane lesion, presence of hyaline, granular or hematic cylinders, diffuse endothelial lesions, vascular engorgement and diffuse micro-thrombosis were some of the most frequent secondary post-traumatic findings reported by these authors. Moon^[8]^ and Mallory's^[9]^ descriptions reported a clinical picture of global, diffuse organic deterioration, in association to typical MOFS histopathological findings. Coincidentally, these histopathological findings were the same as reported afterwards, in cases of MOFS.

Maire and Patton^[10]^, in 1956, reported the development of multiple organic lesions in rats submitted to pre-optic nuclei stereotaxic electrolytic experimental lesions. This paper was the basis for Oliveira et al.^[11]^ experimental methods, to conjecture on the hypothesis of the occurrence of a trauma-induced multiple-system derangement pathophysiologic process, that they called secondary post-traumatic syndrome, possibly based on a central nervous system (CNS)^[12,13]^ imbalance. These authors proposed, based on the Wiener's Theory of Systems logic, that non-specified trauma would result in an adaptation Selye's alarm reaction (AR) CNS-induced syndrome^[13]^. For this reason, in this experimentation, it will be utilized a similar micro-neurosurgical experimental model of MOFS induction.

MOFS can be defined as a complex, subacute, pathological manifestation, necessarily developed after a gap of apparent clinical stability, to a generalized picture of multiple organic deterioration, characterized by confluent anatomic lesions, typically associated to ischemia/reperfusion lesions, finally translated into diffuse micro-thrombosis and tissue hemorrhages^[1]^. MOFS may occur in any severe trauma, such as burns, pulmonary aspiration, multiple transfusions, acute pancreatitis, extracorporeal circulation, sepsis, among many other causes^[1,13-16]^. It is important to reinforce that sepsis is only one of the possible causes of activation of several humoral, inflammatory, coagulation, immunological factors, inducing an average of 35% of all MOFS cases. The remaining 65% of cases are probably based on AR CNS-induced pathophysiology^[1,13,16]^.

### Possible Beneficial Actions of Nitric Oxide in MOFS Development

In 1987, Ignarro et al.^[13]^ detected that the endothelium-derived relaxing factor (EDRF), described in 1980 by Furchgott and Zawadzki^[14]^, was, indeed, a very simple molecule: nitric oxide, that proved, however, to have several important physiologic actions. Such actions, mediated by cyclic guanosine monophosphate (cGMP), can be listed as:

a) regulation of vascular tone, influencing tissue blood flow and arterial pressure;
b) platelet function, by controlling its adhesion and aggregation;
c) neurotransmission at the level of some motor neurons of the parasympathetic branch of the autonomic nervous system that modulates intestinal peristalsis and penile erection;
d) also acts at nitric oxide-sensitive neurons of the respiratory centers of medulla oblongata;
e) nitric oxide (NO) stimulates NMDA receptors at hippocampal area, improving mechanisms of long-term memory;
f) activation of immunity/inflammatory systems, aiding to kill various pathogens, such as virus and bacteria;
g) inhibition of inflammation and exocytosis of various mediators, from endothelial cells, macrophages and cytotoxic T lymphocytes;
h) increase of renal blood flow, with improvement of rate of filtration and urine formation;
i) stimulating endocrine secretions, such as hypothalamic release of gonadotropin-releasing hormone (GnRH);
j) adrenaline from adrenals or other exocrine secretions, such as amylase from the pancreas^[17-23]^.

NO is a labile lipid soluble gas synthesized in several areas of the organic system. Is synthesized from the amino acid l-arginine by the NO synthase (NOS). During this synthesis, NO is transformed into a monomer that, in the presence of tetrahydrobiopterin, turns into a dimer. Therefore, in the presence of calmodulin and molecular oxygen, it converts l-arginine to NO, and citrulline, as a by-product^[22,24,25]^.

Ischiropoulos et al.^[18]^, in 1994, and Preiser et al.^[19]^, in 1995, were the first to suggest the therapeutic utilization of inhaled NO in prophylaxis and treatment of adult respiratory distress syndrome, the most important component of MOFS pathologic constellation. In addition, it was reported that inhaled NO-induced improvement in the pulmonary hypertension associated with experimental septic shock in pigs, with concomitant elevation in the cardiac ejection fraction.

It is important to emphasize that inhaled NO has very short half-lives varying between 6 and 40 seconds^[22,24,25]^. However, when released into the intrapulmonary spaces and pulmonary vessels, NO is inactivated very quickly by combination with hemoglobin, yielding methemoglobin as an end-product. Therefore, the important vasodilator effect of inhaled NO is rapidly neutralized, and remains restricted to the lung vascular and airways systems, with reduced or no systemic effects. In addition, it is very important to emphasize that most of NO systemic effects are due to the activation of cGMP that, on the other hand, is neutralized by 11 different phosphodiesterases. Phosphodiesterase-5 (PDE5) has the most desirable characteristics to be inhibited if secondary vascular and coagulation profile are to be preserved for a longer period. Therefore, if systemic actions are intended, as the needed in MOFS, it must be used a PDE5 inhibitor, that will maintain the NO-induced cGMP activation functioning for a long period, in the entire organic system^[22,26]^.

Therefore, it became obvious that, instead of utilizing inhaled NO, a short half-life gas^[22,24,25]^, the most effective way to universally elevate tissues levels of NO would be through the utilization of PDE5 inhibitors, such as tadalafil, sildenafil, vardenafil, udenafil or avanafil^[22,27]^. Tadalafil would be the best PDE5 inhibitor candidate to inhibit NO degradation and, hence, enhancing the system NO circulating levels, for a prolonged period. NO, indeed, has several beneficial actions, especially the systemic vasodilatation, and reduction of platelet aggregation, that would possibly reduce the development of micro-thrombosis - the MOFS basic inducing factor^[22,24]^. Therefore, it was decided to ascertain the potential protective effects of tadalafil, in the MOFS development, utilizing an experimental neurosurgical model in this syndrome induction^[22]^.

The choice of tadalafil was pharmacologically based on its easy and fast absorption, favorable pharmacokinetic profile, such as low first-pass degradation, large volume of distribution, and relatively slow metabolism and excretion^[28,29]^. In addition, and specially for its long half-life and benign therapeutic profile, it was finally chosen as the drug that, for its characteristics of inducing long-term NO tissue concentrations, would result in of vasodilation and reduction of the coagulation profile^[15,16,20,24,30]^. This would act reducing the potential of development of micro-thrombosis, the pathologic basis for MOFS development. For this reason, the authors proposed to experimentally verify its efficacy in reducing MOFS development, especially by prolonging its multiple-system half-life through the utilization of a PDE5 inhibitor: tadalafil.

## METHODS

### Animals, Environment and General Procedures

The *in-vivo* experiments protocol was approved after a review done by a specific institutional animals experimental research committee and was performed on Wistar male rats weighing from 180 to 220 g that were kept in separate cages, placed in a thermally stable room (21ºC), with free access to water and balanced food. The group ideal size of 10 animals, utilized in this work, was established basing on the statistical previous calculations using experimental groups of 6, 10, 15 and 20 animals. These animals were submitted to the following experimental procedures:

### Microneurosurgical Procedures

The animals were anesthetized with ethyl ether (anesthesia wakening time of 9 minutes + 3 minutes) and placed in a stereotaxic neurosurgical frame (David Kopf 1404, São Paulo, SP, Brazil). After appropriate antisepsis, the parietal bones were exposed and holes (0.5 mm of diameter) were drilled bilaterally in specific places. The Surgical Group followed specifically these procedures: stereotaxic electrolytic lesions (EL) were placed in the anterior hypothalamus (AH) nuclei; by using an anodal constant current of 5 mA for 20 seconds through a monopolar stainless-steel insulated electrode (0.3 mm diameter and 30-tip diameter) previously calibrated in an optical microscope. The stereotaxic parameters were extracted from the König and Klippel stereotaxic atlas^[12]^. Sham-operated (S) animals were submitted to the same procedures, except for the placement of hypothalamic lesions. The electrode was stained with methylene blue for further histological lesion precision placement evaluation.

### Pharmacological Procedures

### The animals were divided into four groups:

#### Control Group

This group was composed by 10 animals, which were injected intraperitoneally with 2 mL of NaCl 0.9%. Twenty-four hours after, these animals were sacrificed through abdominal vessels section, under ethyl ether anesthesia.

#### Surgical Control Group

Composed by 10 animals submitted to the above mentioned protocol of stereotaxic electrolytic lesions^[1]^ under ethyl ether anesthesia. These animals received 2 mL of NaCl 0.9% intraperitoneally. These animals were sacrificed 24 hours after the surgical procedure.

#### Tadalafil 2 Hours after Surgery Group

Composed by 10 animals submitted to the same above mentioned stereotaxic electrolytic procedure, under ethyl ether anesthesia. A solution of tadalafil (Ely Lilly Co., Indianapolis, IN, USA) 0,6 mg/kg dissolved in 2 mL of a solution of NaCl 0.9% was injected intraperitoneally 2 hours after the placement of the hypothalamic lesions. These animals were sacrificed 24 hours after the surgical procedure.

#### Tadalafil 4 Hours after Surgery Group

Composed by 10 animals submitted to the same above mentioned stereotaxic electrolytic procedure under ethyl ether anesthesia. A solution of tadalafil (Ely Lilly Co., Indianapolis, IN, USA) 0.6 mg/kg dissolved in 2 mL of a solution of NaCl 0.9% was injected intraperitoneally 4 hours before the hypothalamic lesions placement. These animals were sacrificed 24 hours after the surgical procedure.

#### Tadalafil 8 Hours after Surgery Group

Composed by 10 animals submitted to the same above mentioned stereotaxic electrolytic procedure, under ethyl ether anesthesia. A solution of tadalafil (Ely Lilly Co., Indianapolis, IN, USA) 0.6 mg/kg dissolved in 2 mL of a solution of NaCl 0.9% was injected intraperitoneally 8 hours after the placement of the hypothalamic lesions. These animals were sacrificed 24 hours after the surgical procedure.

### Histopathology Procedures

The animals were sacrificed 24 hours after the neurologic stereotaxic electrolytic procedure, under ethyl ether anesthesia, by sectioning the abdominal vessels. In our laboratory experience, this period was enough to allow the development of intermediate-to-severe gravity MOFS pattern. In addition, intermediate-to-severe levels of multiorgan involvement would make possible the evaluation of, either attenuation or potentiation of MOFS evolution. Aorta washing was not performed in order to preserve the already formed microthrombosis in several organs. The following organs were removed, fixed in neutral 10% formaldehyde solution and after embedded in paraffin and stained with hematoxylin-eosin for further histological examination: brain, lungs, heart, spleen, liver, kidneys (and adrenals), and stomach-duodenum. Spleen, duodenum, heart and adrenals histology were not studied in this work due to its minimal MOFS-related deterioration. Animals with inappropriately placed cerebral lesions were discarded and replaced in the experimental series. MOFS evolution will be presented as follows: the histopathological assessment was quantitated by reviewing ten microscopic field of every slide, the following an arbitrary grading scale, basing on literature and to our previous experience. The gravity scores varied from 0 to IV.

The histopathology examination was performed by a "blind" physician (pathologist). The microscopic slides were identified only by a computer-generated randomized numbers. The identifications of the slides utilized a code, that was broken after finished slide examination. The scores were attributed to every slide, basing on the sum of each grade of ten 64X microscopic neighbor fields. Therefore, the minimum field damage score (absence of lesion) would be 0 (zero) and the maximum field damage score would be 4 (four). Since were examined 10 fields per slide, that multiplied by each field damage scores, would reach 40 per each organ, reaching 00 or 40. This value, multiplied by 5 organs, would reach a maximal value of 200, and then multiplied by 10 slides per group, we would have a final minimal score of 0000, and a maximum score of 2000. A 250X magnification was utilized, in cases of doubt, only in order to detect specific qualitative tissue damage for grading classification.

### Statistical Analysis

The results (mean±SD) evaluates organ damage through the consolidated gravity scores (CGS) for each experimental setting of n=10 animals, being 00 the baseline and 40 the maximal gravity values. We utilized the Kruskal-Wallis nonparametric statistical method for the overall groups under study. For specific comparisons between two groups, were utilized the χ^2^ method. It were considered significant values of *P*<0.05, or highly significant values *P*<0.01.

## RESULTS

**Fig. 1:**
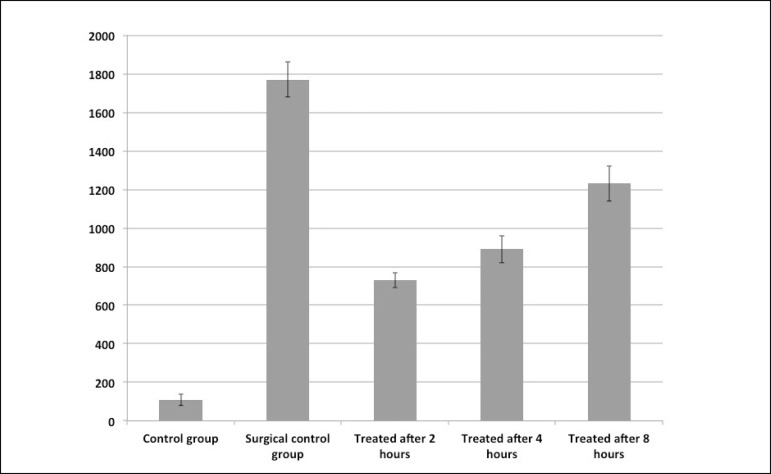
Consolidated gravity scores comparisons. A) Control group compared to surgical control group: P<0.01; B) surgical control group compared to treatment 2 hours after surgery group: P<0.01; C) surgical control group compared to treatment 4 hours after surgery group: P<0.01; D) surgical control group compared to treatment 8 hours after group: P<0.05.

The results of the CGS are presented in Figure 1 and Table 1:

a) control:110±30;
b) surgical control group: 1770±90;
c) 2 hours after tadalafil treatment of surgical group: 730±40, which represents a reduction of 58.75% of the surgical CGS;
d) 4 hours after tadalafil treatment of surgical group: 890±70, which represents a reduction of 49.71% of the surgical CGS;
e) 8 hours after tadalafil treatment of surgical group: 1230±90, which represents a reduction of 30.50% of the surgical CGS.

**Table 1:** Consolidated gravity scores (CGS) comparisons between different experimental groups.

Experimental groups	1) Control group	2) Surgical control group	3) Treatment 2 hours after surgery group	4) Treatment 4 hours after surgery group	5) Treatment 8 hours after surgery group
CGS	X±SD = 110±30	X±SD = 1770±90	X±SD = 730±40	X±SD = 890±70	X±SD d=1230±90
CGS comparison: *P* values		Groups 1 and 2 *P*<0.01	Groups 2 and 3 *P*<0.01	Groups 2 and 4 *P*<0.01	Groups 2 and 5 *P*<0.05

## DISCUSSION

Virchow's^[30]^ classical work, published in 1853, proposed the mechanisms for the development of tissue infarcts, the most important inducing-factor of MOF:

a) low blood flow;
b) endothelial lesion;
c) hypercoagulability.

This study gave to Oliveira et al.^[11]^ the basis for the proposal of peripheral pathophysiologic core of MOFS.Therefore, the following AR-associated peripheral factors would be responsible for the MOFS induction process. As previously considered, generalized microthrombosis is the core lesion in all organs compromised in that syndrome. Then, the sequence of peripheral factors participating in the MOF pathophysiology, as proposed by de Oliveira et al.^[11]^, would be:

- Phase of vasospasm, thrombosis and ischemia:
  - Factor of vasoconstriction and low flow.
  - Factor of endothelial lesion.
  - Factor of hypercoagulability.
- Phase of reperfusion, failure and vascular necrosis:
  - Factor of vascular metabolic deterioration.
  - Factor of vascular hypo-response, dilatation, reperfusion and hyperpermeability.
  - Factor of vascular necrosis and microhemorrhage.

As mentioned before, the three most important PDE5 inhibitors currently available, tadalafil, sildenafil and vardenafil, have a number of functional differences. Especially concerning their selectivity and specificity of inhibition, the PDE5 inhibitors have their effects associated, not only to their safety profile, but also in the biopharmaceutical and pharmacokinetic characteristics. Those differences may, significantly, affect the efficacy profile of these drugs^[15,16,20,28,29]^.

Tadalafil protective actions are, possibly, supported by its cGMP-induced vasodilation, platelet aggregation inhibition and inflammatory modulation effects^[15-17]^. Tadalafil presents absorption of about 36% of the oral dose through the gastrointestinal tract, which is, in general, similar to sildenafil and vardenafil. However, CYP3A is the major hepatic metabolizing enzyme for the 3 PDE5 inhibitor agents, having a lower effect on tadalafil, characteristic responsible for its longer half-life^[13,15-17,28,29]^.

The major route of elimination of all PDE5 inhibitors is hepatic metabolism through CYP3A. Tadalafil, however, has low hepatic extraction. Renal elimination of the nonmetabolized drug represents less than 1% of the elimination pathways. The elimination half-life is 3-5 hours for sildenafil and vardenafil, as compared to 17.5 hours of tadalafil^[13,15-17,28,29]^.

For its greater therapeutic window, tadalafil requires less time for effectiveness, which was one of the most important reasons for its choice as the central drug for testing PDE5 inhibitors as potential protectors against MOF development.

The results presented in this work, shows, definitely, that tadalafil acts protectively against experimentally-induced MOFS development, especially when the drug is administered before the neurosurgical induction procedure. In our experiments, the group of tadalafil treatment done 2 hours after operation resulted in a highly significant (*P*<0.01) reduction of 58.75% of MOFS organ damage gravity. In the group of tadalafil treatment done 4 hours after operation, it was also detected a highly significant (*P*<0.01) reduction of 49.71% of MOFS organ damage gravity. Finally, in the group of tadalafil treatment done 8 hours after operation, it was detected a still significant (*P*<0.05) reduction of 30.50% of MOFS organ damage gravity. The increasing fall in tadalafil protective profile, sequentially detected, among the three post-surgery groups, would probably due to the spontaneously developing MOFS reactions that continues to occur in the not-protected periods of 2, 4 and 8 hours. However, the drug continues to show its protective characteristics, even eight hours after the neurosurgical MOFS induction. Therefore, it suggests that the protective tadalafil effect continue to occur even after a relatively long period after trauma (8 hours), a situation that usually occurs in clinical conditions, such as in clinical emergency-treated cases.

MOFS mortality, regardless whether induced by sepsis or by other etiologies, in the ICUs^[2,3,14-16,22]^ has been between 23% and 76%, whereas, the overall mortality of MOFS patients in hospital environment is between 32% and 89%. Therefore, our experimental work suggests that further clinical trials should be designed to verify tadalafil efficacy in MOFS prophylaxis and treatment in ICU clinical environment.

## CONCLUSION

The results presented in this work, shows, that tadalafil acts protectively against experimentally-induced MOFS development, especially when the drug is administered before the neurosurgical induction procedure.

**Table:** 

Authors' roles & responsibilities	
GGO	Analysis and/or data interpretation; conception and design study; manuscript redaction or critical review of its content; realization of operations and/or trials; statistical analysis; final manuscript approval
SAHO	Conception and design study; manuscript redaction or critical review of its content; realization of operations and/or trials; final manuscript approval
PHHB	Analysis and/or data interpretation; manuscript redaction or critical review of its content; final manuscript approval
MABO	Manuscript redaction or critical review of its content; final manuscript approval
KB	Analysis and/or data interpretation; conception and design study; final manuscript approval
FM	Conception and design study; final manuscript approval
